# Comparative analysis of affected and unaffected areas of systemic sclerosis skin biopsies by high-throughput proteomic approaches

**DOI:** 10.1186/s13075-020-02196-x

**Published:** 2020-05-07

**Authors:** Paraskevi Chairta, Paschalis Nicolaou, Kleitos Sokratous, Christine Galant, Frédéric Houssiau, Anastasis Oulas, George M. Spyrou, Marta E. Alarcon-Riquelme, Bernard R. Lauwerys, Kyproula Christodoulou

**Affiliations:** 1 Cyprus School of Molecular Medicine, 6 Iroon Avenue, 2371 Nicosia, Cyprus; 2 Neurogenetics Department, Cyprus Institute of Neurology & Genetics, 6 Iroon Avenue, 2371 Nicosia, Cyprus; 3 Bioinformatics ERA Chair, Cyprus Institute of Neurology & Genetics, 6 Iroon Avenue, 2371 Nicosia, Cyprus; 4 Present Address: OMass Therapeutics, The Schrödinger Building, Heatley Road, The Oxford Science Park, Oxford, OX4 4GE UK; 5 Department of Pathology, Université catholique de Louvain, Bruxelles, Belgium; 6 Rheumatology Department, Cliniques Universitaires Saint-Luc, Pôle de Pathologies Rhumatismales Inflammatoires et Systémiques, Université catholique de Louvain, Bruxelles, Belgium; 7 Area of Medical Genomics, Pfizer–Universidad de Granada–Junta de Andalucía de Genómica e Investigación Oncológica (GENyO), Parque Tenológico de la Salud Fundación (PTS) Granada, Spain; Arthritis and Clinical Immunology, Oklahoma Medical Research Foundation, Oklahoma City, USA

**Keywords:** Systemic sclerosis, Scleroderma, Biomarkers, Proteomics, Mass spectrometry, Rheumatology, Human, Skin biopsy

## Abstract

**Background:**

Pathogenesis and aetiology of systemic sclerosis (SSc) are currently unclear, thus rendering disease prognosis, diagnosis and treatment challenging. The aim of this study was to use paired skin biopsy samples from affected and unaffected areas of the same patient, in order to compare the proteomes and identify biomarkers and pathways which are associated with SSc pathogenesis.

**Methods:**

Biopsies were obtained from affected and unaffected skin areas of SSc patients. Samples were cryo-pulverised and proteins were extracted and analysed using mass spectrometry (MS) discovery analysis. Differentially expressed proteins were revealed after analysis with the Progenesis QIp software. Pathway analysis was performed using the Enrichr Web server. Using specific criteria, fifteen proteins were selected for further validation with targeted-MS analysis.

**Results:**

Proteomic analysis led to the identification and quantification of approximately 2000 non-redundant proteins. Statistical analysis showed that 169 of these proteins were significantly differentially expressed in affected versus unaffected tissues. Pathway analyses showed that these proteins are involved in multiple pathways that are associated with autoimmune diseases (AIDs) and fibrosis. Fifteen of these proteins were further investigated using targeted-MS approaches, and five of them were confirmed to be significantly differentially expressed in SSc affected versus unaffected skin biopsies.

**Conclusion:**

Using MS-based proteomics analysis of human skin biopsies from patients with SSc, we identified a number of proteins and pathways that might be involved in SSc progression and pathogenesis. Fifteen of these proteins were further validated, and results suggest that five of them may serve as potential biomarkers for SSc.

## Introduction

Systemic sclerosis (SSc), also known as scleroderma, refers to a systemic rheumatic disease that is generally classified as an autoimmune disease (AID) [1, 2]. It is characterised by the three main pathological hallmarks: vasculopathy, immune system abnormalities and excessive deposition of collagen (fibrosis) in many tissues throughout the human body causing hardening and thickening [1, 3]. This disease is heterogeneous and multisystemic as symptoms vary among patients and several organs of the human body might be affected [3].

Based on its clinical features, SSc is subdivided into limited cutaneous SSc (lcSSc) and diffused cutaneous SSc (dcSSc) [4]. LcSSc is less severe than dcSSc as skin thickening is usually limited to the distal extremities, finger, upper neck and face. However, in some cases, lcSSc patients present mild organ-complications. In contrast to lcSSc, dcSSc progresses faster and usually affects internal organs (e.g. lung, kidney and heart) causing complications such as congestive heart failure, renal crisis and interstitial lung disease (ILD). In addition, serological data showed that different autoantibodies are produced in each subtype [3, 4]. Anti-centromere autoantibodies (ACA) are mainly present in lcSSc patients, whereas anti-topoisomerase (ATA) and anti-RNA-polymerase (ARNAP) autoantibodies in dcSSc patients [5].

Up to date, SSc aetiopathogenesis is not well understood; thus, its prognosis and diagnosis are challenging and there is no cure for the disease [6]. Therefore, there is an urgent requirement for SSc biomarkers that could be used not only for prognosis and diagnosis of the disease, but also for SSc staging, activity, classification and for potential therapeutic targets. During the last two decades, proteomics biomarker discovery has been developed due to the advances of mass spectrometry (MS) approaches. MS, a high-throughput technique, enables the identification and quantification of proteins in a variety of biological samples such as saliva, plasma and serum [7].

The aim of this proteomic study was to analyse affected and unaffected skin biopsy samples from patients with SSc in order to identify biomarkers and pathways which are implicated in SSc pathogenesis. We hereby report some associated pathways and the validation of possible SSc proteomic biomarkers.

## Materials and methods

### Clinical samples

Fourteen paired cutaneous biopsies were obtained from clinically affected (forearm) and clinically unaffected (proximal arm) skin from seven patients with SSc, voluntarily participating in the PRECISESADs project (ref: 115565) following the appropriate written informed consent procedures. The study was approved by the Ethical Committee of Université catholique de Louvain (2014/17DEC/603) and the Cyprus National Bioethics committee (ΕΕΒΚ/ΕΠ/2015/31). All patients fulfilled the LeRoy and Medsger criteria [8] and were in the active phase of the disease.

### Histological classification of skin biopsy samples

Histological features of SSc skin biopsies (collagen bundles and inflammatory cells levels) were assessed.

Evaluation of optical parameters (density of collagen bundles and inflammatory cell infiltrates) was performed using a semi-quantitative score on a 0–3 scale, where 0 indicates absence, 1 weak and very focal staining, 2 moderate and focal staining, and 3 moderate in several foci. Perivascular fibroblastic densification was assessed as present or not [9].

### Pre-analytical sample processing

Frozen skin biopsies were cryo-pulverised to a fine powder using a cell crusher device (Cellcrusher, Cork). Skin powdered samples were suspended in a lysis buffer (10 mM Tris-HCl pH 7.4; 150 mM NaCl; 1 mM EDTA; PBS; 0.2% SDS; Proteinase Inhibitor (Roche, USA)) and homogenised by sonication. Then, protein precipitation was performed using 1 ml of frozen acetone at − 20°*C*, overnight. Protein pellet was re-suspended in urea buffer, and total protein content was determined by the BCA assay. A total of 100 μg of protein was transferred to centrifugal filter units (30 kDa MWCO, Pall NanoSep Omega, OD030C34, Sigma Aldrich), and a modified filter-aided sample preparation (FASP) protocol [10] was followed. Briefly, proteins were reduced with 8 mM dithiothreitol at 56°*C* for 15 min, alkylated with 50 mM iodoacetamide at room temperature for 20 min in the dark and then digested with trypsin (Proteomics Grade, Roche Life Science, USA) at 1: 50 (trypsin: protein) ratio for 18 h at 37°*C*. Tryptic peptides were collected by centrifugation, and trifluoroacetic acid (TFA) was added to a final concentration of 0.5% to stop enzymatic reaction. TFA concentration was reduced to 0.1%, and peptides were purified and desalted by solid phase extraction using Sep-pak®Vac1cc (100 mg) tC18 cartridges (Waters, Ireland). The eluted peptides were dried, using centrifuge vacuum at 45°*C* and stored in − 80 °C until further analysis.

### LC/MS methods

Dried peptide pellets were re-suspended in buffer (99% H_2_O, 1% acetonitrile, 0.1% formic acid). The total peptide concentration was determined by absorption at 280 nm (A280) and was adjusted accordingly to a final concentration of 0.2 μg/μl prior liquid chromatography-mass spectrometry (LC-MS) analysis. For the discovery phase, 2 μl was loaded onto an analytical column (nanoAcquity CSH C18 75 μm ID X 250 mm length, 1.7 μm particle size, 130 Å pore size, Waters, UK) and separated on a nano-liquid chromatography (LC) system (nanoAcquity UPLC, Waters, UK) at a flow rate of 0.300 μl/min using a 220-min gradient elution. Column oven temperature was set to 40°*C*. Eluted peptides were ionised in positive mode using nanoelectrospray ionisation (nanoESI) and analysed on a Waters Synapt G2Si HDMS instrument operated in ion mobility mode, using an ultra-definition (UD) MS^e^ approach [11].

For the validation phase, the MRM (Multiple Reaction Monitoring) analyses were performed using specific selected analytes. In the first step, a pool of selected synthetic peptides (2.5 μg) was loaded onto the analytical column and separated as descripted in the discovery phase using a 125-min gradient elution and the column oven temperature was set to 45°*C*. For the skin biopsy samples, 10 μl of 0.8 μg/μl total peptide concentration were loaded. Peptides were analysed on a Waters Xevo TQD instrument operated on positive MRM mode.

### Protein identification and quantification and statistical analyses (discovery phase)

Raw MS data were processed by the Progenesis QI for proteomics (QIp) analysis software. Peptide identifications were performed using the MS^e^ [12] search identification, with 1% peptide false discovery rate (FDR). The resulted identifications were further refined using the following parameters: confidence score ≥ 5, sequence length ≥ 6 and hits ≥ 2. Protein-level relative quantitation was performed using the Hi-N approach (*N* = 3) as implemented in the Progenesis QIp. Further statistical analysis was carried out according to affected/unaffected paired samples (all affected versus all unaffected skin biopsy samples), and this is referred to as affected/unaffected comparison in this manuscript. A variation of one-way ANOVA analysis, as implemented in the Progenesis QIp software, was used to calculate *p* values. Data normalisation was performed by Progenesis QIp software, using the ‘normalise to all compounds’ option. Briefly, the normalisation approach is based on the calculation of a global scaling factor which is then applied to normalise samples to an automatically selected reference sample.

### Selection of proteins for the validation phase

Selection of proteins for validation was performed based on *p* value and FC through the affected/unaffected comparison. Significantly differentially expressed proteins (*p* < 0.05) with FC > 2.5 or < 0.4 in affected/unaffected comparison were selected.

### Synthesised peptides for validation

For the validation phase, selection of target peptides for proteins quantification was performed using the Skyline 4.1 source (MacCross Lab software, University of Washington, WA, USA) [13]. At least two unique peptides were selected for each protein of interest. Synthesised label-free peptides (~ 70% purity) were purchased from GenScript Biotech, Netherlands. The targeted MS method parameters used for each peptide are shown in Additional file 1.

### MRM-MS data and statistical analyses

The transition list of targeted peptides was created by the Skyline 4.1 source. The peak areas of peptides were calculated by summing the peak areas of their transition ions.

Statistical analysis of validation data was carried out using two-tailed paired *t* test for paired samples analysis (all affected versus all unaffected skin biopsy samples). A *p* value of < 0.05 was considered as significant. For each protein, the peptide with the strongest *p* value was indicated as quantitative, while the rest were used as qualitative references. The FC was calculated by comparing the mean value of the peak areas of the peptide among the grouped samples.

### Bioinformatics tools

The *Uniprot Retrieve/ ID mapping tool* (http://www.uniprot.org/uploadlists/) was used to convert the UniProtKB AC/ID to Gene Name.

*Pathway analysis* was performed for significantly over-expressed (*p* < 0.05, FC > 1.5) or under-expressed (*p* < 0.05, FC < 0.67) proteins from affected/unaffected comparison in the discovery phase. Enrichr (http://amp.pharm.mssm.edu/Enrichr/) was utilised for inferring pathway knowledge about the gene set corresponding to the differentially expressed proteins, based on KEGG 2019.

*Volcano plots* were carried out using the R studio software (version 1.1.442, 2018-03-11). The volcano plot indicates the -log_10_ (*p* value) for proteins plotted against their respective log_2_ (fold change (FC)) on the *y* and *x* axes, respectively.

## Results

### Skin biopsy samples

Five female and two male unrelated SSc patients were analysed with a mean ± SD age of 56 ± 15.80 years and 38.5 ± 9.20 years, respectively. Skin biopsies were histologically assessed, and their features are shown in Additional file 2.

### Discovery proteomic analysis

Discovery phase proteomic analysis of the analysed SSc samples led to the identification and quantification of 2149 proteins. Samples were grouped and compared based on affected/unaffected skin biopsy areas. This comparison showed that 169 (87 under-expressed and 82 over-expressed) out of 2149 identified proteins were significantly differentially expressed (*p* < 0.05) (Additional file 3) (Table 1).

**Table 1:** Discovery and validation results from affected/unaffected comparison selected proteins

Protein	Discovery ***p*** value (FC)	Synthesised peptides	Validation ***p*** value (FC)
P05090|APOD	0.03 (2.55)	**VLNQELR**	0.22 (1.51)
		NILTSNNIDVK	D
P43897|TSFM	0.04 (0.35)	GFLNSSELSGLPAGPDR	D
		**TNLEDVGR**	0.15 (4.37)
		LGQHVVGMAPLSVGSLDDEPGGEAETK	D
P62266|RPS23	0.01 (0.36)	**AHLGTALK**	0.10 (2.39)
		VANVSLLALYK	D
P30740|SERPINB1	0.04 (0.38)	**TYNFLPEFLVSTQK**	0.46 (0.66)
		TYGADLASVDFQHASEDAR	D
		LGVQDLFNSSK	D
P09936|UCHL1	0.00 (2.52)	LGVAGQWR	D
		LGFEDGSVLK	D
		**NEAIQAAHDAVAQEGQC[+57]R**	**0.01 (2.62)**
P02743|APCS	0.02 (2.58)	VGEYSLYIGR	D
		**IVLGQEQDSYGGK**	**0.05 (5.54)**
Q53GQ0|HSD17B12	0.00 (0.37)	**TIAVDFASEDIYDK**	0.41 (0.58)
		GVFVQSVLPYFVATK	D
Q08752|PPID	0.04 (2.50)	IVLELFADIVPK	N/D
		VFFDVDIGGER	D
		**ILLITEDLK**	**0.02 (1.69)**
Q8NHQ9|DDX55	0.04 (3.33)	DVAAEAVTGSGK	D
		**SLDVLVLDEADR**	**0.03 (4.40)**
		TGLFSATQTQEVENLVR	D
Q15154|PCM1	0.04 (2.98)	**INFSDLDQR**	N/D
		LPEMEPLVPR	N/D
		ALYALQDIVSR	N/D
P14854|COX6B1	0.02 (5.26)	**NC[+57]WQNYLDFHR**	**0.02 (3.73)**
		GGDISVC[+57]EWYQR	D
Q99504|EYA3	0.02 (4.18)	**LSSGDPSTSPSLSQTTPSKDTDDQSR**	0.49 (0.73)
		VLLYGLGEIFPIENIYSATK	D
Q9Y262|EIF3L	0.03 (0.38)	**VYEIQDIYENSWTK**	0.28 (0.64)
		VFSDEVQQQAQLSTIR	D
		LAGFLDLTEQEFR	D

This table shows the selected proteins from affected/unaffected comparison, their synthesised peptides and *p* value and FC from discovery and validation phases. Peptide sequence with bold font indicates the peptides that were used for quantification. The remaining peptides were used as quality controls. Bold font in validation *p* value (FC) column indicates the significant *p* values in the validation phase

*D* detected, *N/D* not detected

### Pathway analysis

Pathway analysis of significantly over-expressed (*p* < 0.05, FC > 1.5) and under-expressed (*p* < 0.05, FC < 0.67) proteins from affected/unaffected comparison revealed a large number of involved pathways. Significant extracted pathways (*p* < 0.05) are shown in Table 2. Several pathways that are associated with SSc pathogenesis were also identified in this study. Platelet activation, ECM-receptor interaction, complement and coagulation cascades, antigen processing and presentation and leukocyte transendothelial migration are among these significant extracted pathways (Table 2).

**Table 2:** Significant extracted KEGG 2019 pathways that are associated with *p* < 0.05, FC > 1.5 or < 0.67 proteins in affected versus unaffected comparison

Pathway	Pathway’s ***p*** value	Pathway	Pathway’s ***p*** value
Hypertrophic cardiomyopathy (HCM)	**1.53E−08**	*Staphylococcus aureus* infection	**2.25E−02**
Dilated cardiomyopathy (DCM)	**2.47E−08**	Adherens junction	**2.50E−02**
Cardiac muscle contraction	**2.61E−07**	Arrhythmogenic right ventricular cardiomyopathy (ARVC)	**2.50E−02**
Adrenergic signalling in cardiomyocytes	**6.10E−07**	Kaposi sarcoma-associated herpesvirus infection	**2.55E−02**
Focal adhesion	**5.90E−05**	Calcium signalling pathway	**2.62E−02**
Regulation of actin cytoskeleton	**8.81E−05**	Bacterial invasion of epithelial cells	**2.63E−02**
Pertussis	**1.30E−04**	Gastric acid secretion	**2.70E−02**
Complement and coagulation cascades	**1.52E−04**	Antigen processing and presentation	**2.83E−02**
Proteoglycans in cancer	**6.09E−04**	Epstein-Barr virus infection	**3.11E−02**
Glycolysis/gluconeogenesis	**1.61E−03**	PI3K-Akt signalling pathway	**3.24E−02**
Phagosome	**1.79E−03**	Human immunodeficiency virus 1 infection	**3.56E−02**
Cellular senescence	**2.16E−03**	Fc gamma R-mediated phagocytosis	**3.84E−02**
cGMP-PKG signalling pathway	**2.47E−03**	Small cell lung cancer	**4.00E−02**
ECM-receptor interaction	**2.75E−03**	Amoebiasis	**4.23E−02;***3.79E−02*
Histidine metabolism	**2.75E−03**	Aldosterone synthesis and secretion	**4.39E−02**
AGE-RAGE signalling pathway in diabetic complications	**4.80E−03**	Pancreatic secretion	**4.39E−02**
Human papillomavirus infection	**5.29E−03**	HIF-1 signalling pathway	**4.56E−02**
Fructose and mannose metabolism	**5.62E−03**	Chagas disease (American trypanosomiasis)	**4.80E−02**
Leukocyte transendothelial migration	**6.58E−03**	Ribosome	*1.72E−15*
Human T cell leukaemia virus 1 infection	**6.59E−03**	Huntington disease	*3.82E−04*
Ferroptosis	**8.17E−03**	Systemic lupus erythematosus	*8.72E−04*
Platelet activation	**8.70E−03**	Spliceosome	*8.97E−04*
Carbohydrate digestion and absorption	**9.83E−03**	Synaptic vesicle cycle	*2.00E−03*
Vascular smooth muscle contraction	**1.03E−02**	Alcoholism	*2.65E−03*
Apelin signalling pathway	**1.14E−02**	Oxidative phosphorylation	*8.91E−03*
Parkinson disease	**1.25E−02;***1.06E−02*	Endocrine and other factor-regulated calcium reabsorption	*1.03E−02*
Mineral absorption	**1.30E−02**	Necroptosis	*1.52E−02*
Cell adhesion molecules (CAMs)	**1.33E−02**	Steroid hormone biosynthesis	*1.58E−02*
Viral myocarditis	**1.72E−02**	Arachidonic acid metabolism	*1.74E−02*
Tight junction	**2.02E−02**	Viral carcinogenesis	*2.66E−02*
Shigellosis	**2.06E−02**		

This table shows the significant extracted pathways that are associated with *p* < 0.05, FC > 1.5 or < 0.67 proteins in affected versus unaffected comparison. Bold and italic fonts indicate pathways that were extracted from *p* < 0.05, FC > 1.5 and *p* < 0.05, FC < 0.67 proteins, respectively

### Selected proteins and validation

**Fig. 1:**
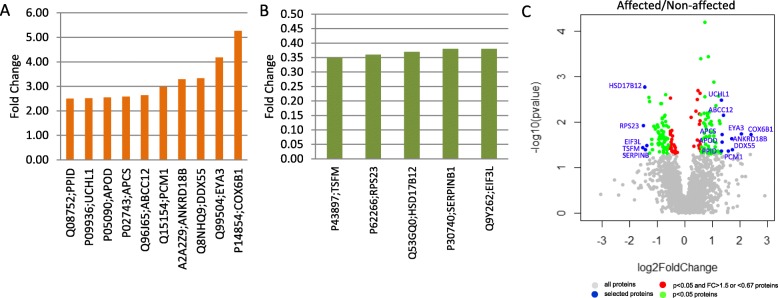
Significantly dysregulated proteins (*p* < 0.05) with FC > 2.5 or < 0.4 in affected/unaffected paired sample comparison. The bar graphs show the significantly dysregulated proteins (*p* < 0.05) with **a** FC > 2.5 and **b** < 0.4 in affected compared to unaffected paired samples. **c** Volcano plot reporting *p* values against FC for affected/unaffected comparison. It indicates -log_10_ (*p* value) for affected/unaffected comparison proteins (*y*-axis) plotted against their respective log_2_ (fold change) (*x*-axis)

Although many of the dysregulated proteins could be promising biomarkers for SSc, a limited number of proteins were selected for validation due to the high cost of MRM-MS approach. A total number of 15 proteins were selected for validation through the affected/unaffected comparison (Fig. 1).

Five (UCHL1, PPID, DDX55, COX6B1 and APCS) out of these proteins were confirmed in the validation phase to be significantly dysregulated in affected/unaffected comparison and all details and results are shown in Table 1. UCHL1 belongs to the ubiquitin C-terminal hydrolases family and enables the hydrolysis of small ubiquitin adducts [14]. PPID (CyP40) is implicated in protein folding, nuclear localisation of progesterone, oestrogen and glucocorticoid receptors, ligand binding, pro-tumorigenic effects and congenital heart defects [15, 16]. DDX55 belongs to the DEAD-box proteins which are implicated in several RNA metabolism processes such as RNA transcription, degradation as well as gene expression in organelles and pre-mRNA splicing [17]. COX6B1 is a subunit of Complex IV and Massa et al. reported that a recessive mutation on *COX6B1* causes a mitochondrial encephalomyopathy [18]. The last confirmed protein is the plasma glycoprotein APCS which functions as a calcium-dependent lectin [19] and is involved in immunological responses [20].

## Discussion

SSc is a multisystemic AID with unclear aetiology and pathogenesis. The main pathological features of the disease, vasculopathy, inflammation and fibrosis, highlight its complexity and indicate that various molecules and pathways are implicated in the different stages of disease pathogenesis. However, these molecules and pathways/mechanisms have not been fully elucidated [21]. Therefore, the aim of this study was to analyse skin biopsies from affected and unaffected areas of the same SSc patients in order to study different stages of the disease and identify sensitive proteomic biomarkers gaining insights into the pathways/mechanisms that are implicated in SSc pathogenesis.

Using discovery MS proteomic analysis of SSc samples, 2149 proteins were identified and 169 proteins were shown to be significantly dysregulated in the affected/unaffected comparison. Pathway analysis of these proteins confirmed the heterogeneity of the disease as the proteins are involved in approximately 190 different pathways. As SSc is an extremely complex disease and several molecules, cell types and biological processes are implicated in its pathogenesis [6], several of the bioinformatics-highlighted pathways might be associated with the disease in different ways. Furthermore, some of these pathways are related with other diseases including AIDs. These findings indicate that several diseases especially AIDs might share common pathogenetic mechanisms. According to the Enrichr analyses, platelet activation, ECM-receptor interaction, complement and coagulation cascades, antigen processing and presentation and leukocyte transendothelial migration are among the common significant extracted pathways.

It is known that overexpression of the ECM proteins plays a key role in the development of fibrosis in SSc [22]. Our data confirm this knowledge as three proteins (ITGB1, VTN and ITGA5) that are implicated in ECM-receptor interaction pathway were over-expressed in the affected/unaffected comparison. Proteins that are implicated in complement and coagulation cascades (C4B, VTN, SERPINC1 and CLU), and antigen processing and presentation pathways (HSPA4 and HLA-G) were also significantly over-expressed in this comparison. Although this observation is consistent with the results obtained in a previous study, these pathways are not only activated in SSc but also in other AIDs [23, 24]. Another important pathway that was extracted from ITGB1, AKT3 and MYLK over-expressed proteins in the affected/unaffected comparison is the platelet activation pathway which contributes to all three stages of SSc pathogenesis: vascular injury, inflammation and fibrosis [25]. In a previous study, Agache et al. reported that platelet activation markers are associated with the severity and activity of the disease and increased levels of C-reactive protein (CRP) [26]. Leukocyte transendothelial migration is also an essential pathway as CD4+ T lymphocytes transendothelial migration is enhanced in this disease and the migrating cells display an activated phenotype [27]. The ITGB1, ACTN1 and ICAM1 proteins that were identified to be significantly over-expressed in affected/unaffected comparison in our study are implicated in this pathway.

As already stated, a large number of proteins were identified; however, only 15 proteins were selected for validation due to the high cost of MS. In the validation phase, 5 (UCHL1, PPID, DDX55, COX6B1 and APCS) out of these 15 selected proteins were further confirmed to be significantly dysregulated. Giusti et al. in a transcriptomic study showed that ubiquitin C-terminal hydrolases protein UCHL1 and other molecules that are implicated in ubiquitination/stress are highly expressed in skin endothelial cells of dcSSc patients compared to controls [14, 28]. In the validation phase of our study, UCHL1 was confirmed to be significantly over-expressed in affected/unaffected comparison. It is remarkable that overexpression of UCHL1 in SSc affected skin areas is confirmed both at the transcriptome and proteome levels by two independent studies. CyP40 is another protein that was found to be significantly over-expressed in the affected/unaffected comparison in our study. Interestingly, Balanescu et al. showed that cyclophilin-A, a member of the same family with CyP40, is abnormally expressed in biological fluids and cutaneous biopsies of SSc patients [7]. These evidences suggest that UCHL1 and CyP40 could be promising SSc biomarkers and further studies on these two molecules should be performed. DDX55 and COX6B1 were also significantly over-expressed in the affected/unaffected comparison. These two proteins have been associated with SSc for the first time in our study; thus, additional investigation should be performed in order to further confirm their association with the development of SSc and their potential use as biomarkers of the disease. APCS is a plasma glycoprotein which is implicated in immunological responses [20]. Tennent et al. used serum of lcSSc and dcSSc patients and 12-month longitudinal patients and showed that APCS levels were in the normal range; except from a limited number of elevated values that are associated with acute inter-current complications [29]. Aden et al. showed that the APCS precursor level was significantly overexpressed in skin biopsies from SSc patients compared to healthy controls [30]. In agreement with Aden et al., our study showed that APCS is significantly over-expressed in the affected/unaffected comparison in both the discovery and validation phases. However, this protein may generally be associated with autoimmunity instead of specifically with SSc as it was found to be implicated in other AIDs as well [31].

This study shows different proteomic background and histological features among affected and unaffected skin biopsies of the same SSc patients. This is the first study that compares SSc skin areas macroscopically classified as affected and unaffected. Despite the importance of the results, the small number of samples used is a limitation of this study. However, previous reported studies also had this limitation [32, 33], because SSc is a rare disease, and thus, large numbers of patients are not expected to be recruited. Although skin biopsies were obtained only from SSc patients and not from healthy controls, this could be also considered as an advantage as the comparison is performed on samples from the same individual and the bias of heterogeneity is reduced.

## Conclusions

In conclusion, the results of this study are important as they display the promising diagnostic power of a multi-biomarker approach. Pathway analysis showed that several pathways are implicated/activated in SSc pathogenesis. Available literature on UCHL1 and PPID proteins supports that they could be promising biomarkers for SSc. Interestingly, 3 (DDX55, PPID and COX6B1) out of 5 confirmed proteins have been associated with SSc for the first time in our study; therefore, these proteins could be novel biomarkers of the disease. In addition, APCS is associated with several AIDs and inflammatory pathways; thus, it might not be specific for SSc. However, they could be used as inflammation-related biomarkers. Further studies could be performed using samples from patients with different AID in order to assess whether these molecules are mainly associated with any other specific AID or with general inflammatory conditions. Further evaluation/validation studies could be performed in samples of new patients with SSc in order to confirm that these 5 molecules could be also useful biomarkers for specific stages (e.g. early phase) of the disease. Moreover, confirmation of these biomarkers in an easily accessible tissue will be useful for the clinicians and alleviate painful procedures for the SSc patients.

## Supplementary information

**Additional file 1.** MRM transitions used for the selected proteins analysis. This table shows the selected proteins/peptides for validation and the instrumental parameters used for peptide monitoring. Peptides highlighted in bold were used as the quantitative reference whereas the remaining peptides were used as qualitative references.

**Additional file 2.** Clinical features of patients with SSc and histological features of SSc skin biopsy samples.

**Additional file 3 **Significantly differentially expressed proteins (*p* < 0.05) in affected/unaffected comparison. Red and green font colour indicates proteins with FC ≥ 1.5 and FC ≤ 0.67 in affected compared to unaffected samples.

## Data Availability

The datasets used and/or analysed during the current study are available from the corresponding author on reasonable request.

## Abbreviations

ACA: Anti-centromere autoantibody
AIDs: Autoimmune diseases
ARNAP: Anti-RNA-polymerase autoantibody
ATA: Anti-topoisomerase autoantibody
CRP: C-reactive protein
DcSSc: Diffused cutaneous SSc
FASP: Filter-aided sample preparation
FC: Fold change
FDR: False discovery rate
ILD: Interstitial lung disease
LC: Liquid chromatography
LC-MS: Liquid chromatography-mass spectrometry
LcSSc: Limited cutaneous SSc
MRM: Multiple reaction monitoring
MS: Mass spectrometry
nanoESI: Nanoelectrospray ionisation
PBMCs: Peripheral blood mononuclear cells
SLE: Systemic lupus erythematosus
SSc: Systemic sclerosis
TFA: Trifluoroacetic acid
UD: Ultra-definition
UPS: Ubiquitin-proteasome system
USPs: Ubiquitin-specific peptidases
